# COVID-19 and inequities in the Americas: lessons learned and implications for essential health services

**DOI:** 10.26633/RPSP.2021.130

**Published:** 2021-12-28

**Authors:** Anselm J.M. Hennis, Anna Coates, Sandra del Pino, Massimo Ghidinelli, Rodolfo Gomez Ponce de Leon, Edwin Bolastig, Luis Castellanos, Renato Oliveira e Souza, Silvana Luciani

**Affiliations:** 1 Pan American Health Organization Washington, D.C. United States of America Pan American Health Organization, Washington, D.C., United States of America

**Keywords:** Health status disparities, effective access to health services, COVID-19, noncommunicable diseases, risk factors, social determinants of health, Americas, Disparidades en el estado de salud, acceso efectivo a los servicios de salud, COVID-19, enfermedades no transmisibles, factores de riesgo, determinantes sociales de la salud, Américas, Disparidades nos níveis de saúde, acesso efetivo aos serviços de saúde, COVID-19, doenças não transmissíveis, fatores de risco, determinantes sociais da saúde, América

## Abstract

The COVID-19 pandemic has exacerbated social, economic, and health-related disparities, which disproportionately affect persons living in conditions of vulnerability. Such populations include ethnic groups who face discrimination and experience barriers to accessing comprehensive health care. The COVID-19 pandemic has exposed these health disparities, and disruptions of essential health services have further widened the gaps in access to health care. Noncommunicable diseases are more prevalent among groups most impacted by poor social determinants of health and have been associated with an increased likelihood of severe COVID-19 disease and higher mortality. Disruptions in the provision of essential health services for noncommunicable diseases, mental health, communicable diseases such as HIV, tuberculosis, and malaria, and maternal and child health services (including sexual and reproductive health), are projected to also increase poor health outcomes. Other challenges have been an increased frequency of interpersonal violence and food insecurity. Countries in the Americas have responded to the disruptions caused by the pandemic by means of health service delivery through telemedicine and other digital solutions and stepping up social service support interventions. As vaccinations for COVID-19 create the opportunity to overcome the pandemic, countries must strengthen primary health care and essential health services with a view to ensuring equity, if the region is to achieve universal health coverage in fulfillment of the Sustainable Development Goals.

## RESUMEN

La pandemia de COVID-19 ha acentuado las desigualdades sociales, económicas y relacionadas con la salud, que afectan desproporcionadamente a las personas en situación de vulnerabilidad. Esta población incluye grupos étnicos que se enfrentan a la discriminación y obstáculos para el acceso a la atención integral de salud. La pandemia de COVID-19 ha expuesto estas desigualdades de salud, y las interrupciones de los servicios esenciales de salud han ampliado aún más las brechas en el acceso a la atención de salud. Las enfermedades no transmisibles son más prevalentes en los grupos que han sufrido un mayor impacto de los determinantes sociales de la salud deficientes y se han asociado con una mayor probabilidad de presentar un cuadro grave de COVID-19 y una mayor mortalidad. Asimismo, se proyecta que las interrupciones en la prestación de servicios esenciales de salud para las enfermedades no transmisibles, la salud mental, las enfermedades transmisibles como la infección por el VIH, la tuberculosis y la malaria, y los servicios de salud maternoinfantil (como la salud sexual y reproductiva) incrementen los resultados deficientes en materia de salud. Otros retos son una mayor frecuencia de la violencia interpersonal y la inseguridad alimentaria. Los países de la Región de las Américas han respondido a las interrupciones causadas por la pandemia con la prestación de servicios de salud mediante la telemedicina y otras soluciones digitales, y la aceleración de las intervenciones de apoyo de los servicios sociales. A medida que la vacunación contra la COVID-19 crea la oportunidad de superar la pandemia, los países deben fortalecer su atención primaria de salud y sus servicios de salud esenciales a fin de garantizar la equidad, para que la Región logre la cobertura universal de salud en cumplimiento de los Objetivos de Desarrollo Sostenible.

## RESUMO

A pandemia de COVID-19 exacerbou as disparidades sociais, econômicas e as relacionadas à saúde, que afetam de maneira desproporcional as pessoas que vivem em situação de vulnerabilidade. Essas populações incluem grupos étnicos que enfrentam discriminação e barreiras para o acesso à atenção integral à saúde. A pandemia de COVID-19 expôs essas disparidades, e as interrupções nos serviços essenciais de saúde ampliaram ainda mais as lacunas no acesso aos cuidados de saúde. As doenças não transmissíveis são mais prevalentes entre os grupos mais afetados por determinantes sociais da saúde deficientes e estão associadas a um aumento na probabilidade de doença grave pela COVID-19 e mortalidade mais elevada. Prevê-se que as interrupções na prestação de serviços essenciais de saúde para doenças não transmissíveis, saúde mental, doenças transmissíveis como HIV, tuberculose e malária, bem como dos serviços de saúde materno-infantil (incluindo saúde sexual e reprodutiva) também aumentem os desfechos adversos de saúde. Outros desafios são o aumento da frequência da violência interpessoal e insegurança alimentar. Os países das Américas responderam às interrupções causadas pela pandemia com a prestação de serviços de saúde por meio da telemedicina e outras soluções digitais, e a aceleração de intervenções de apoio dos serviços sociais. À medida em que a vacinação contra a COVID-19 oferece a oportunidade de superar a pandemia, os países devem fortalecer a atenção primária à saúde e os serviços essenciais de saúde com o objetivo de garantir a equidade, para que a região atinja a cobertura universal de saúde em cumprimento aos Objetivos de Desenvolvimento Sustentável.

The Region of the Americas manifests marked economic, social, and health disparities. In Latin America and the Caribbean, the richest 10% earn 22 times more than those in the bottom 10% (1). Socioeconomic, gender, ethnic, and structural inequities underpin reduced life expectancy, higher childhood and maternal mortality, poor nutrition, mental health conditions, and both noncommunicable and communicable diseases (2, 3). Gender inequalities also underpin risk of interpersonal violence (4). Social and structural determinants, resulting in poor housing and workplace conditions, overcrowded living circumstances, constrained transportation, and inequities in access to quality health services, particularly affect ethnic groups who face discrimination (5, 6). These factors and limited access to services also constrain appropriate pandemic responses (7). These scenarios have also been complicated by major population movements, such as mass emigration from Venezuela to surrounding countries.

The COVID-19 pandemic has had significant adverse impact in the Region of the Americas, with more than 90.2 million cases and 2.2 million deaths, by 1 October 2021, accounting for 38.7% and 46.6% of global cases and deaths, respectively (8). This article reviews the impact of the COVID-19 pandemic on priority health outcomes, particularly in vulnerable populations in Latin America and the Caribbean, as well as strategies and interventions used by countries to mitigate the negative impact. Finally, lessons learned from country experiences are used to inform approaches to strengthen essential health services that were significantly disrupted during the pandemic.

Information was obtained through a review of the literature conducted between March 2020 and June 2021 using the following keywords: social determinants of health; Latin America and the Caribbean; United States of America; Canada; noncommunicable diseases; mental health; communicable diseases; violence; nutrition; sexual and reproductive health; and COVID-19; as well as through a review of relevant gray literature.

## SOCIAL DETERMINANTS AND PRIORITY HEALTH ISSUES

The leading causes of death and disability in the Americas are noncommunicable diseases (NCDs), mental health conditions, and violence and injuries (9). Disparities and social inequities underpin NCDs, which account for eight in every ten deaths. While an estimated 250 million people live with at least one underlying chronic condition (10), these conditions are not equally distributed across populations. The lowest income quintile, where Indigenous and Afro-descendant populations are overrepresented, is associated with the highest rates of obesity, hypertension, and uncontrolled diabetes. This situation has been exacerbated by the differences in quality of and access to health services, depending on health system financing and maturity, participation in social security schemes, access to private health insurance and private care systems, and health literacy and access to health information (7).

Social determinants are known to underpin mental health conditions, and in the Americas there is a tremendous gap between the number of people who need mental health care and those who receive it. This so-called treatment gap is much higher in Latin America and the Caribbean than in North America (11). Women and children are vulnerable to interpersonal violence, with nearly one-third (29.8%) of ever-partnered women in Latin America and the Caribbean having been physically and/or sexually abused by an intimate partner, and the pandemic has exacerbated this situation (4).

## ACCESS TO NUTRITION

The COVID-19 pandemic has worsened food insecurity, and a recent study estimated that 75.7% of persons in Latin America and the Caribbean were food insecure, with populations in Venezuela, Nicaragua, and Haiti being most affected (12). Poor diets high in ultraprocessed foods coupled with physical inactivity during lockdowns are likely to worsen COVID-19 outcomes.

## DISRUPTIONS IN ESSENTIAL HEALTH SERVICES

The COVID-19 pandemic has led to redirection of scarce resources and disruptions in already weak and fragmented health systems. A rapid assessment of health services in 2020 in the Region of the Americas demonstrated major disruptions to services for NCDs, particularly diabetes and hypertension management, as well as to mental health services (13), at a time when the need for such services has been high. A follow-up survey beginning five months later indicated that disruptions remained highest in the Region of the Americas, at around 50%, compared with the other five regions of the World Health Organization (14). Services for communicable diseases such as HIV, tuberculosis, and malaria were also disrupted.

Reproductive health services have also been drastically impacted by the COVID-19 pandemic, where significant barriers already existed as a result of determinants such as education, health literacy, and poor reproductive health care access (15). It has been estimated that the proportion of women with unmet family planning needs in Latin America will increase from 11.4% to as much as 17.7%, resulting in 1.7 million unplanned pregnancies, around 800 000 abortions, 2 900 maternal deaths, and 39 000 infant deaths as a consequence of the pandemic, unless appropriate interventions are implemented to ensure access to quality services (16).

## VULNERABLE POPULATIONS MOST AFFECTED

A year after the start of the COVID-19 pandemic, a pattern of rapid spread of the SARS-CoV-2 virus affecting large numbers was evident in the United States of America, Brazil, Mexico, Colombia, Peru, Argentina, Canada, Bolivia, and the Dominican Republic, where the daily cases exceeded 1 000 affected persons. In the United States of America, Black and Hispanic groups not only experienced higher rates of COVID-19 but also a three- to six-fold higher mortality than comparable White groups. Factors included higher prevalence of comorbidities such as obesity and NCDs, known to increase the likelihood of severe COVID-19-related illness and death (17). Additionally, these groups are overrepresented among front-line health workers who are at higher risk of exposure. Studies from Latin America described impact on COVID-19 rates due to low socioeconomic status, overcrowding, using public transport, living in slums, having limited access to clean water, reliance on boat travel, and informal work being the source of income (18–20). The risk of dying from COVID-19 has also been shown to be higher in Indigenous peoples, those with subsidized health insurance, and those of low socioeconomic status (21).

## STRATEGIES TO MINIMIZE IMPACT ON ESSENTIAL HEALTH SERVICES

COVID-19 has exposed structural deficiencies in health, social, and economic policies, and countries have responded with interventions to mitigate the impact. Social interventions have included: unemployment grants; food transfers; donation of cleaning and hygiene supplies; reduced utility rates; cash transfers; prevention of dismissal from work; delayed tax filings; protection from eviction; debt relief; suspension of credit payments; and rental forgiveness (22). However, as noted by Benítez et al. (23), “the economic support measures put in place were found to be too timid for some countries and introduced too late in most of them.”

Regarding nutritional challenges, there needs to be special focus on ensuring availability of and access to nutritious foods and promoting sustainable food production. This strategy should ensure the continuity of essential nutrition and early child development services and promote investments in social protection systems and programs with particular attention to groups with nutritional vulnerability.

Health service disruptions have been compensated for by providing phone consultations, self-management support, electronic prescriptions, and extending the period length of prescriptions, among other strategies. However, in the post-pandemic period, essential health services will require significant scaling up, with access especially for NCD care, mental health support, sexual health education and services, and health literacy, while prioritizing vulnerable populations. Violence prevention will require the implementation of strategies with the inclusion of violence prevention in pandemic preparedness and response plans and in risk mitigation communications, with adequate resource allocation. These strategies must be aimed especially at women, children, and older adults; agencies and professionals developing and implementing the policies; and be based on information, prevention, supporting survivors, and working across sectors. Additionally, these strategies must be linked to data collection.

Telehealth strategies have become more important as a tool to support social distancing. However, there are major gaps, with broadband access available to only around 50% of the population of Latin America and the Caribbean (24). Significant investment in infrastructure as well as the development of supporting legislation will be required to advance this innovation, which has the potential to scale up delivery of care in the post-pandemic recovery period.

## LESSONS LEARNED

Latin America is characterized by heterogeneous health systems, which include private care, social security systems, and publicly funded systems operated by ministries of health serving lower-income population groups. In the context of the COVID-19 response, decentralized health services functioning at the level of states, municipalities, or provinces, or social insurance organizations have further increased fragmentation and constrained an integrated and coordinated health response. Furthermore, chronic underinvestment in health has led to weak public primary health care and hospital systems, in turn resulting in widespread pandemic-related health system and service disruptions.

We have learned of the importance of mandatory public health and disease control prevention policies, including population lockdowns, quarantines, and curfews. However, it is clear that social protection mechanisms, access to health services, and non-pharmacological interventions should have been scaled up at the same time, to support socioeconomically vulnerable populations with poor social determinants, who have often been most affected by the SARS-CoV-2 virus. This approach would identify communities at risk for active inclusion in the health net and for targeted social support, based on health outcome data indicating populations with highest disease burdens and greatest barriers to accessing health services.

If countries are going to advance toward universal health coverage and achieve the Sustainable Development Goals, major investments will be required in health systems and services, with a scaling up of human resources for health. The emphasis must be on a strengthened primary health care system.

Strategies to eliminate barriers to health access in this time of the pandemic might include provision of mobile services or deployment of community health workers or health brigades to such communities. Interventions that worked have included extending the period of prescriptions and even making medications available in community centers to reduce the need for visits to congregate facilities.

Migrants in Latin America and the Caribbean face particular socioeconomic vulnerabilities and are often outside the social protection net with limited or no access to health care. Social policies at the national level must be strengthened to be inclusive of these groups, particularly during this time.

The Pan American Health Organization posits that, “the resilience of health systems and societies will partly depend on countries’ capacity in three main areas”: (1) realignment of core values—which will shift societal core values toward health, social cohesiveness, and development, with inclusive and sustainable economic development; (2) prioritization and investment in health, social development, and protection; and (3) transformation and investment in health systems to ensure preparedness for external threats, while simultaneously maintaining universal access to health and universal health coverage (universal health) (25).

## CONCLUSION

The COVID-19 pandemic has highlighted the need for countries to adequately invest in their health systems and services to prevent the level of disruptions seen in this pandemic from occurring in future health emergencies. As vaccines for COVID-19 become available, there must be strengthening of primary health care with a view to ensuring equity. It is also critical to include responses to preserve and maintain functioning essential health services in the national health emergency response plans. In the specific national contexts, emergency responses must include interventions to safeguard the rights of those in vulnerable situations who face the greatest risks in terms of both health sector responses and wider social policy responses.

## Disclaimer.

Authors hold sole responsibility for the views expressed in the manuscript, which may not necessarily reflect the opinion or policy of the *Revista Panamericana de Salud Pública/Pan American Journal of Public Health* and/or those of the Pan American Health Organization.
